# Prevalence of Myocardial Infarction in Saudi Arabia: A Systematic Review

**DOI:** 10.7759/cureus.64761

**Published:** 2024-07-17

**Authors:** Tariq M Shaqran, Renad S Almutairi, Elaf J Zurayyir, Sally AlOlayan, Hassan Salamah Alfuhaid, Fatema Sayed Ali Alalawi, Huda A Al-Haddad, Husain Y Buhasan, Janan J Husain, Fatema M Isa, Batool A Mahdi

**Affiliations:** 1 Family Medicine, King Salman Armed Forces Hospital, Tabuk, SAU; 2 College of Medicine, King Abdulaziz University Hospital, Jeddah, SAU; 3 Faculty of Medicine, Jazan University, Jazan, SAU; 4 Family Medicine, Qassim Health Cluster, Qassim, SAU; 5 Internal Medicine, King Salman Specialist Hospital, Hail, SAU; 6 Family Medicine, Salmaniya Medical Complex, Manama, BHR; 7 College of Medicine, Southeast University (SEU), Nanjing, CHN; 8 College of Medicine, Soochow University, Suzhou, CHN

**Keywords:** coronary artery disease, cardiovascular disease, saudi arabia, heart attack, myocardial infarction

## Abstract

Myocardial infarction (MI), frequently referred to as a heart attack, happens when the blood supply to a region of the myocardium is reduced. It might be quiet or devastating, causing hemodynamic decline and rapid death. The most common cause of MI is coronary artery disease, which is the leading cause of mortality in the United States. Prolonged lack of oxygen can lead to myocardial cell loss and necrosis. Patients may report chest pain, pressure, and electrocardiogram alterations. Management of MI relies greatly on the interprofessional team. The purpose of this study was to determine the incidence of MI in Saudi Arabia. Between 2000 and 2024, English-language papers were gathered to demonstrate the prevalence of MI in Saudi Arabia. Overall, there were four articles. Surveys and studies of national databases were the most utilized methods (n=4). We found that heart attacks are a significant health issue in Saudi Arabia, with certain lifestyle choices and medical conditions increasing the risk. Heart attacks are a major health concern in Saudi Arabia. To lower the number of heart attacks, it's important for people to make healthier lifestyle choices.

## Introduction and background

Myocardial infarction (MI), sometimes known as a heart attack, occurs when blood flow to a portion of the myocardium is diminished. It might be modest or severe, resulting in hemodynamic deterioration and sudden death. The most prevalent cause of MI is coronary artery disease, which is the leading cause of death in the United States. Prolonged oxygen deprivation can cause myocardial cell loss and necrosis. Patients may experience chest tightness or pressure, and myocardial ischemia can be linked with ECG abnormalities and elevated metabolic markers. INTERHEART, an international multi-center case-control research study, discovered adjustable risk variables for coronary artery disease (CAD), highlighting the close connection between myocardial infarction and the disease [1,2].

The INTERHEART research discovered that smoking, abnormal lipid profile, hypertension, diabetes, abdominal obesity, psychosocial variables, lack of daily fruit or vegetable consumption, lack of physical exercise, and alcohol use were all strongly related with acute myocardial infarction. Alcohol intake had a lesser correlation, but smoking and an aberrant apolipoprotein ratio had the greatest relationship. Women were more likely to develop diabetes and hypertension, and they also had a greater protective benefit from exercise and alcohol. These risk variables were shown to be strongly linked with acute myocardial infarction [3].

Several factors lead to MI, including a significantly high homocysteine in the blood level, which is a risk factor by itself. This may be treated with vitamin B6, folic acid, or B12 supplements. Non-modifiable components include older age, male gender, and genetics, with the function of genetic loci in increasing MI risk being thoroughly investigated [4].

MI incidence varies by demographic in the US, with non-Hispanic Whites, non-Hispanic Blacks, Hispanics, and non-Hispanic Asians having the highest rates. Between the years 2005 and 2014, the Atherosclerosis Risk Factor in Communities Study (ARIC) predicted 605,000 new MIs and 200,000 recurrent MIs. The first MI typically strikes a man at age 65.6 and a woman at age 72.0. That being said, statistics indicate that MI is becoming less common in the US [5].

Severe blockage of the major epicardial coronary arteries for more than 20-40 minutes can produce a myocardial infarction, a thrombotic blockage caused by plaque rupture. This results in a lack of oxygen in the heart, causing disruption of sarcolemmal cells and myofibril relaxation. This is the first ultrastructural alteration observed in MI, then follow mitochondrial modifications. Prolonged ischemia causes liquefactive necrosis of cardiac tissue to spread through the subendocardium into the subepicardium, resulting in increased collateral circulation. Cardiac function is impaired, and the infarcted area heals with scar formation. The heart regularly remodels, causing dilation, segmental hypertrophy, as well as cardiac dysfunction [6].

Ischemia of the myocardium is a condition in which the equilibrium of oxygen supply and need is interrupted, resulting in abnormal blood flow. This can result in myocardial infarction, which can be detected using a combination of clinical features, electrocardiographic findings, and elevated blood biomarkers. Symptoms of myocardial ischemia comprise chest pain, upper extremity pain, mandibular or epigastric pain, breathlessness or fatigue, perspiration, nausea, stomach pain, dyspnea, and syncope. Myocardial ischemia is assessed using three criteria: clinical features, ECG deviations, and cardiac biomarkers. The resting 12-lead ECG is the main diagnostic test for an acute coronary syndrome (ACS), which should be obtained after 10 minutes of the patient's arrival at the emergency department. Acute MI is typically associated by dynamic changes to the ECG waveform, and serial ECG monitoring may provide useful clues to the diagnosis if the first EKG is non-diagnostic upon presentation [7-9].

ECG anomalies that suggest continuous artery blockage include ST-segment elevation in both contiguous leads, depressed ST-segment and T-wave changes, and hyperacute T-wave amplitude. Other ECG markers of myocardial ischemia included cardiac arrhythmias, intraventricular blocks, atrioventricular conduction delays, and a decrease in precordial R-wave amplitude. Cardiovascular troponins (I and T) are contractile machinery components in myocardial cells found virtually exclusively in the heart. Elevated blood levels of cardiac troponin are unrelated to the kind of damage. An acute MI is characterized by a rising and/or decreasing trend in cardiac troponin (cTn) levels, with at least one result surpassing the 99th percentile of the upper reference limit (URL), and is linked to myocardial ischemia. Serial measurements of cTn levels at 0 hours, three hours, and six hours would offer a better understanding of the severity and persistence of myocardial injury [10,11].

Imaging techniques are used to evaluate myocardial perfusion, viability, thickness, thickening, and motility, as well as the impact of myocyte loss on the kinetics of paramagnetic and radio-opaque contrast agents that detect myocardial fibrosis or scarring. The imaging modalities that can be used include echocardiography, radionuclide imaging, and cardiac magnetic resonance imaging (MRI). Echocardiography can promptly identify regional wall motion abnormalities caused by ischemia when more than 20% of the transmural myocardial thickness is affected, even shortly after the onset of ischemia [10].

The most often used analgesics for pain treatment are intravenous opioids like morphine, which may be linked to an increased risk of death and poor clinical results. However, studies have identified no significant negative effects associated with morphine use in cases of anterior ST-segment elevation MI. Individuals suffering from a myocardial infarction should receive supplemental oxygen in addition to pain treatment. Intravenous nitrates are more helpful than sublingual nitrates for alleviating symptoms and reversing ST depression. Beta-blockers, which reduce myocardial oxygen consumption by lowering heart rate, blood pressure, and myocardial contractility, are recommended for their effectiveness. Platelet suppression is also recommended for ST-elevation myocardial infarction (STEMI) and non-STEMI (NSTEMI) [8,12].

Clopidogrel, prasugrel, and ticagrelor are common P2Y12 inhibitors that suppress thromboxane A2 production throughout the platelet life cycle. Patients undergoing percutaneous coronary intervention (PCI) should get dual antiplatelet therapy (DAPT), which consists of aspirin, a P2Y12 inhibitor, and a parenteral anticoagulant. PCI anticoagulants include unfractionated heparin, enoxaparin, and bivalirudin. Long-term treatment for MI includes lipid-lowering medications, antithrombotic therapy, antihypertensive therapy, and glucose-lowering therapy. Smoking cessation is the most cost-effective secondary therapy for avoiding MI since it causes thrombosis and is associated with atherosclerosis and myocardial infarction. A diet low in saturated fat and high in whole grains, vegetables, fruits, and seafood is regarded to be cardioprotective [13,14].

The mortality rate for acute MI varies between 5 to 30%, with most deaths occurring prior to hospital admission. Diabetes, advanced age, delayed reperfusion, low ejection fractions, congestive heart failure, elevated C-reactive protein (CRP) and brain natriuretic peptide (BNP) levels, and depression all contribute to a poor prognosis. MI consequences include ischaemia, reinfarction, infarction extension, angina, arrhythmias, mechanical, myocardial dysfunction, cardiac failure, cardiac rupture, embolism, inflammation, pericarditis, and pericardial effusion. Myocardial infarction is a complex and varied condition that requires a combination of medicine, lifestyle modifications, and lifestyle improvements. The extent of cardiac muscle injury and ejection fraction dictate the prognosis, with better outcomes for those who preserve left ventricular function [15,16]. Therefore, the objective of this systematic review is to investigate the prevalence of myocardial infarction

## Review

Materials and methods

Search Strategy

To systematically collect studies relevant to our study issue, we used a multimodal search method. This entailed searching key databases including PubMed, Scopus, Web of Science, and the Cochrane Library. Our search included research published up to 2024. To maximize the retrieval of relevant articles, we developed a search algorithm that combined Medical Subject Headings (MeSH) phrases with free-text words. The key search phrases were "myocardial infarction," "heart attack," "MI," "coronary artery disease," and "Saudi Arabia." These terms were merged with the Boolean operators "AND" and "OR" to include comparable keywords and phrases. For example, our PubMed search string was as follows: ("Myocardial infarction" OR "MI"), ("Heart attack" OR "coronary artery disease"), and "Saudi Arabia".

To cover a larger range of relevant literature, we widened our search to include regional databases like the Saudi Medical Literature Database and Index Medicus for the Eastern Mediterranean Region. We then manually reviewed the reference lists of the indicated papers and pertinent reviews for other research that may have been overlooked during the database search.

Inclusion and Exclusion Criteria

Our systematic evaluation included a wide range of papers on the prevalence of myocardial infarctions in Saudi Arabia. We evaluated observational studies of various types as well as cross-sectional studies. Inclusion criteria were poor left ventricular function (ejection fraction (EF) < 35%), severe coronary disease, and viability in four faulty myocardium areas that can be revascularized with PCI. Our goal was to collect data from various contexts, both hospital and community-based, with no restrictions on publication date or language, and to translate non-English papers for thorough analysis.

On the other hand, our exclusion criteria were designed to ensure the scientific validity of our review. We excluded myocardial infarction that occurred less than four weeks before randomization (clinical definition), decompensated heart failure with inotropic support, invasive or non-invasive ventilation, or intra-aortic balloon pump (IABP)/left ventricular assist devices (LVAD) medication within 72 hours after randomization, sustained ventricular tachycardia (VT)/ventricular fibrillation (VF) or tolerable implantable cardiac defibrillator (ICD) discharges within 72 hours before randomization, valve illness that requires intervention, contraindications for PCI, age < 18 years, and estimated glomerular filtration rate (eGFR) < 25 ml/min (unless on dialysis); women who are pregnant; previously participated in REVIVED-BCIS2 or is presently enrolled in another research that may influence REVIVED-BCIS2 outcome data, and life expectancy is less than one year owing to non-cardiac pathology. Furthermore, studies that lacked critical characteristics such as sample size, diagnostic techniques, or prevalence rates were omitted since the authors were unable to provide them. Finally, non-peer-reviewed sources, such as grey literature and editorials, were eliminated to maintain data integrity.

Study Selection

Our research selection strategy was meticulously planned to ensure complete identification and evaluation. Initially, all identified articles were loaded into EndNote to aid duplication removal. The original screening procedure involved two independent reviewers thoroughly analyzing study titles to eliminate unneeded or noncompliant studies. These reviewers then thoroughly examined the abstracts to assist us in narrowing down our options even further.

Articles deemed appropriate at this point underwent a full-text review by an additional pair of independent examiners to confirm they met our criteria. This detailed investigation ensured that the technique, demographics, and outcomes were thoroughly evaluated. Discrepancies at any stage were resolved by conversation or through involving a third reviewer. A comprehensive search of references in selected publications was also conducted to ensure that all essential information was included.

Data Collection

Our data extraction method was carried out with precise accuracy and rigorous rigour. This work was assigned to two separate reviewers, who used a unique data extraction form created specifically for this review. This form collected critical data elements such as research characteristics, design, setting, population demographics, diagnostic procedures for myocardial infarction, and primary findings. In situations of data extraction discrepancies, reviewers met for consensus talks, with a third reviewer making an objective opinion as needed. This phase guaranteed that data was collected accurately and consistently.

Results

Article Screening and Selection

**Figure 1:**
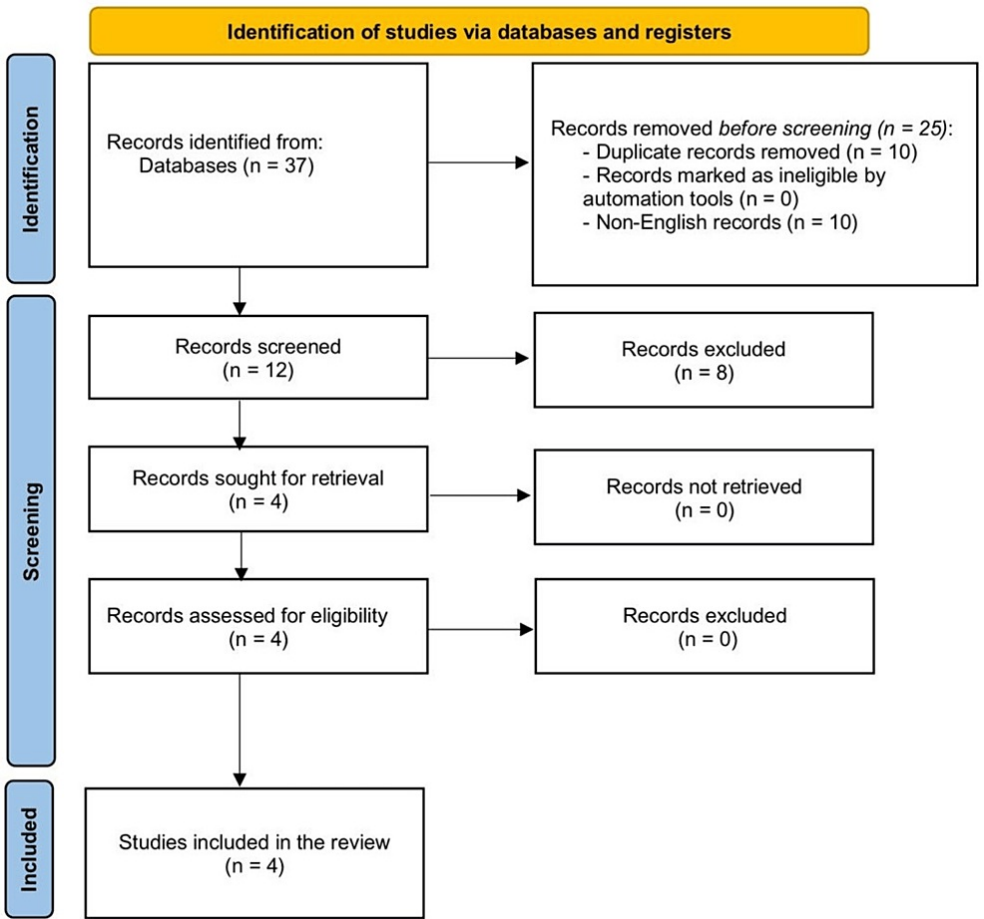
Flow diagram describing the article selection and screening procedure in the studies

In the initial part of our systematic examination, we did a thorough search and identified 37 relevant publications. Following deduplication, 30 unique articles remained. After a rigorous screening, 19 articles were removed due to irrelevance, lack of research material, or deviation from the topic. The other nine publications were subjected to a thorough full-text review, and four studies were chosen for our meta-analysis based on strict inclusion criteria for quality and relevance. The Recommended Reporting Items for Systematic Reviews and Meta-Analyses (PRISMA) [17] criteria were followed, as indicated in Figure 1.

Data Extraction

Table 1 provides a brief description of the data, which includes the author(s) name and year, study aim, and finally the conclusion of the study.

**Table 1:** Brief Description of the Data GFR: glomerular filtration rate; RR: relative risk; OR: odds ratio; CI: confidence interval; MI: myocardial infarction; PCI: percutaneous coronary intervention; STEMI: ST-elevation myocardial infarction; ACS: acute coronary syndrome; EMS: Emergency Medical Services

Serial no.	Author and year	Aim of the study	Conclusion
1	Alharbi et al., 2022 [18]	The purpose of this research was to determine how often stroke and myocardial infarction occur in people whose glomerular filtration rate (GFR) has decreased (GFR < 60 mL/min).	Stroke constituted 2.4% of the total medical cases. Relative risk (RR) (95% CI) = 2.1371 (0.9804-4.6584), p = 0.0561, for the risk of stroke linked with deteriorating GFR. Male prevalence was 3.7% while female prevalence was 1.2%. Myocardial infarctions occurred in 3.2% of the population, with an incidence of 4.7% in men and 2% in women.
2	Alsaab et al., 2023 [19]	This study's overarching goal is to ascertain what variables contribute to the general Saudi public's lack of knowledge of myocardial infarction (MI) symptoms and risk factors.	Nearly 90% of those who took the survey identified chest discomfort and almost 90% of people who reported shortness of breath as symptoms of myocardial infarction. A hefty proportion of people were aware of the risks associated with smoking (90.3%) and obesity (76.1%). On average, participants knew about 26.6±7.5 symptoms and risk factors, and about 36% had this knowledge. Both risk variables and symptom awareness were positively linked with higher levels of education, but employment status was negatively associated with both. Greater knowledge of risk variables was associated with higher income. Men nevertheless showed less knowledge of symptoms and risk factors, which was a major finding. Risk variables and symptom awareness were strongly correlated with region and chronic disease status. Upon recognizing a MI attack, almost 90% of subjects felt compelled to travel to the emergency department without delay.
3	Alahmadi et al., 2020 [20]	The goals of this study are(1) to identify variables related with pre-hospital delay and(2) to assess the amount of time patients diagnosed with acute myocardial infarction spend waiting for treatment.	A median of 3.7 hours elapsed before patients reached the hospital. The median time from the beginning of symptoms and the patient's arrival at the hospital was 126 minutes, or 63% of the total. Arrival by ambulance (adj OR=0.3, 95% CI 0.1-0.8), history of hypercholesteremia (adj OR=2.3, 95% CI 1.1-4.7), prior information on acute coronary syndrome (adj OR=0.35, 95% CI 0.1-0.6), and increased pain intensity (adj OR=0.7, 95% CI 0.6-0.9) were all factors that were significantly associated with pre-hospital delay.
4	Alhabib. et al., 2019 [21]	Our objective was to assess the clinical features, treatment, and results of a statistically valid group of Saudi Arabian patients who had suffered an acute myocardial infarction (AMI).	The 2233 patients with ACS who were included between May 2015 and January 2017 had a mean age of 56 years (standard deviation = 13), were 85.7% male, 55.6% Saudi nationals, and 65.9% had STEMI. Predisposing variables for coronary artery disease were considerable, including hypertension (51.2%) and diabetes mellitus (52.7%) among the participants. On a mere 5.2% of occasions EMS were dispatched. Thrombolytic treatment (29%), primary percutaneous coronary intervention (PCI) (42.5%), pharmaco-invasive approaches (3%), and neither (29%), were the methods used for revascularization in patients with STEMI. Primary percutaneous coronary intervention (PCI) was less common among non-Saudis with STEMI than among Saudis (35.8% vs. 48.7%; respectively, p <0.001), and women were less likely than males to reach a door-to-balloon time of less than 90 minutes (42% vs. 65%; respectively, p = 0.003). About 50% of individuals who had a non-ST-elevation myocardial infarction did not get a coronary angiography. In-hospital, one-month, and one-year rates of all-cause death were 4%, 5.8%, and 8.1%, respectively. Compared to men, these rates were far greater for women.

Discussion

Our systematic review examines the prevalence of myocardial infarction in Saudi Arabia, focusing on four carefully selected studies. The research highlights the growing health issue of myocardial infarction, a growing concern due to increasing cases worldwide and specifically within Saudi Arabia. Saudi Arabia, an economically powerful nation with a rapidly growing economy, is suffering from "an illness of civilization" or "Lifestyle Disease," a group of noncommunicable diseases (NCDs) including cancer, obesity, hypertension, acne, and diabetes. In accordance with the National Institute of Health Metrics and Evaluations, 16.4% of Saudis have a risk of dying from NCDs and the resulting life years adjusted for disability lost (DALYs) [22].

MI is a severe condition resulting from oxygen deficiency, leading to myocardial cell necrosis and potentially fatal hemodynamic deterioration. Acute MI affects 130,974 Saudis annually, and the number of people with coronary heart disease (CHD) is increasing. Direct medical costs are expected to triple from $272.50 billion in 2010 to $818.1 billion by 2030. Hospitals aim to reduce hospitalizations to save money, but this limits the effectiveness of education. Identifying patients' learning needs is crucial for improving time allocation. CVDs are the largest cause of mortality globally, accounting for 17.9 million deaths per year [23].

A study of 3079 STEMI patients found that 16% were women, 70% were from Middle Eastern countries, and 39% were non-Arabic speakers. Women were older, had lower smoking, and had a history of revascularization. However, there were no significant differences in post-MI complications, management, or hospital outcomes between genders and ethnic groups [21]. Saudi Arabia prioritizes early detection and diagnosis of MI through a comprehensive protocol that includes clinical assessments and diagnostic testing. Patients are advised to seek medical help immediately for symptoms like chest discomfort and shortness of breath, following established guidelines for timely treatment [24].

Our studies included in the review have demonstrated that MI is a significant health issue in Saudi Arabia, with its prevalence influenced by various factors and gender. Early detection and diagnosis are crucial, with a comprehensive protocol involving clinical assessments and diagnostic testing. Risk factors include smoking, family history, and hypercholesterolemia for individuals under 45, while diabetes and hypertension are more common among those 45 and over. Smoking and dyslipidemia are common risk factors in both age groups. Although older individuals have a higher mortality rate, the elderly and young people have comparable mortality rates but reduced morbidity. The study found a significant frequency of MI in Saudi Arabia, ranging from 47.8% to 72.8%, indicating the severity of the problem and the potential contribution of genetic predispositions, food choices, and lifestyle behaviors to its high frequency [20]. Research shows smoking, family history, and hypercholesterolemia are the most common risk factors for people under 45, while diabetes and hypertension are more common in patients over 45. Older individuals have a higher fatality rate from MI, while younger individuals have lower morbidity [25].

A cross-sectional survey by Alharbi et al. 2022 found a 2.4% stroke prevalence in Saudi participants, with a higher risk associated with deteriorated GFR. The prevalence was higher in males (3.7%), females (1.2%), middle-aged adults (4.7%), and older individuals (4.7%). Myocardial infarctions were also more prevalent in males (4.7%). The study concluded that a deteriorated GFR <60 mL/min is significantly associated with stroke and myocardial infarction [19]. In contrast, a survey by Mohan et al. 2018 revealed that 63% of patients arrive late at the hospital, higher than in industrialized nations (51.4%) but lower than in developing countries (67.7%-81%). The median pre-hospital delay was 3.7 hours, like in Saudi Arabia and Malaysia. The study discovered no significant differences between men and women. Other nations observed median pre-hospital delays of two hours in Turkey, 2.5 to four hours in Sweden, and 2.5 to four hours in China [25].

Several factors were discussed in a previous study which demonstrated that Saudi Arabia's obesity rate is 49.6%, with dyslipidemia, hypertension, and diabetes at 32.1% and 25.2% respectively. This is due to the country's rapid economic expansion, poor diet, and less physical activity. In 2018 a poll showed 82.6% of Saudis were sedentary, and 88% consumed less than the recommended five servings of fruits and vegetables daily. Two-thirds of the population is obese or overweight [26]. In addition, patients sustaining a stroke were treated with caution owing to bleeding concerns. Most patients are active or former smokers, have high lipid levels, or are obese [27]. Older age, male gender, overweight, hypertension, smoking, diabetes, hypertriglyceridemia, and hypercholesterolemia are important causes of MI. Previous studies have demonstrated that management options to lower modifiable risk factors, lifestyle adjustments, and metabolic syndrome treatment are required [28]. Another study by Sheikh et al. found a significant correlation between aging and MI in Saudi Arabia, with smoking being the leading risk factor for both men and women. Risk variables included smoking, diabetes, hypertension, family history of IHD, and hypertension combined with diabetes. Early preventive and intervention adjustments could benefit both genders [29]. Arabia could partially be attributed to regional lifestyle factors, such as high-calorie diets and sedentary habits, which are known contributors to metabolic disorders. These findings emphasize the need for targeted public health strategies focusing on dietary and lifestyle modifications to combat this trend [19].

Finally, our study also highlights the multifaceted nature of myocardial infarction, shaped by genetic, environmental, and lifestyle factors. The elevated incidence of myocardial infarction in Saudi Arabia could partially be attributed to regional lifestyle factors, such as high-calorie diets and sedentary habits, which are known contributors to metabolic disorders. The US is the primary source of non-invasive, widely available, and cost-effective diagnostic methods in reviewed studies, but its lower sensitivity, especially for mild myocardial infarction cases, raises concerns about underestimation of prevalence. Future studies should incorporate more sensitive diagnostic methods like MRI or liver biopsies for more accurate prevalence estimates [30]. The review emphasizes the need for standardized diagnostic criteria and consistent study methodologies in myocardial infarction research. Current variations in methods and designs hinder direct comparisons, necessitating a standardized approach for more precise data and reliable prevalence estimates. This would enable better comparisons across different studies and populations, ensuring more accurate and reliable outcomes [31].

The study emphasizes Saudi Arabia's high myocardial infarction rate and calls for public health interventions focusing on lifestyle modifications, early detection, and effective care. It calls for more research on inclusive population studies and the progression rate of screen-detected myocardial infarction, particularly in type 2 diabetes mellitus (T2DM) contexts, and calls for an integrated approach combining public health initiatives, research, and healthcare practices.

## Conclusions

The study emphasizes the high frequency of myocardial infarction in Saudi Arabia, as well as the complexity of its causes, which are impacted by lifestyle, genetics, and environment. It advocates for strong public health initiatives for prevention and management, particularly in high-risk groups. The study emphasizes the importance of continued research and standardized diagnostic criteria to better understand and respond to this emerging health concern.
